# Changes in the incidence of occupational disability as a result of back and neck pain in the Netherlands

**DOI:** 10.1186/1471-2458-6-190

**Published:** 2006-07-18

**Authors:** Ivan A Steenstra, Jos H Verbeek, Femmeke J Prinsze, Dirk L Knol

**Affiliations:** 1 Coronel Institute for Occupational Health, Academic Medical Center, University of Amsterdam, The Netherlands; 2 Department of Public and Occupational Health, VU University Medical Centre, Amsterdam, The Netherlands; 3 UWV, National Institute for Social Insurance, Amsterdam, The Netherlands; 4 Department of Clinical Epidemiology and Biostatistics, VU University Medical Center, The Netherlands; 5 Body@Work, Research Center Physical Activity, Work and Health, TNO-VU University Medical Center, The Netherlands; 6 Finish Institute of Occupational Health, Kuopio, Finland

## Abstract

**Background:**

Back pain (including neck pain) is one of the most prevalent health problems for which physicians are consulted. Back pain can decrease the quality of life considerably during a great part of the lives of those who suffer from it. At the same time it has an enormous economic impact, mainly through sickness absence and long-term disability. The objective of this paper is to compare the incidence of occupational disability as a result of back and neck pain in 1980–1985 to 1999–2000 and to explain the findings.

**Methods:**

A descriptive study was performed at population level of changes in incidence of occupational disability as a result of back and neck pain. Statistics from the National Institute of Social Insurance in the Netherlands are used to calculate age and gender specific incidence rates for back pain diagnoses based on the ICD-classification. Incidence rate ratios stratified according to gender and adjusted for age were calculated to indicate changes over time.

**Results:**

The incidence of occupational disability as a result of back pain decreased significantly by 37% (95% CI 37%–38%) in men and with 21% (95% CI 20%–24%) in women, after adjustment for age. For overall occupational disability as a result of all diagnoses this was 18% (95% CI 18%–19%) and 34% (95% CI 33%–35%) respectively. Changes were not homogeneous over diagnostic subcategories and age groups. Spondylosis decreased most in men by 59% (95% CI 57%–61%). The incidence of non-specific back pain and neck pain increased most by 196% (95% CI 164%–215%). Post-laminectomy syndrome increased over all age categories both for men (85%, 95% CI 61%–113%) and women (113%, 95% CI 65%–179%).

**Conclusion:**

The decrease in occupational disability as a result of back pain was larger than the decrease in occupational disability over all diagnoses. However, time trends were not homogeneous over age-, nor over sex- nor back pain categories. Most of this decrease was due to general changes such as legal and economic changes. One of several additional explanations for a decrease is the changed view on management of back pain.

## Background

Back pain is one of the most prevalent health problems for which physicians are consulted [1]. Back pain can decrease the quality of life considerably during a great part of the lives of those who suffer from it. At the same time it has an enormous economic impact, mainly through sickness absence and long-term disability [2]. In the past decade, it has become clear that there is the advice to stay active is one of the very few interventions that has effect on back pain in the acute stage [3]. This advice to stay active has been shown to influence sick leave, both in the acute and subacute stage of back pain [4,5]. More comprehensive rehabilitation programs that follow the same principle of activation are reported to be effective [6,7]. In line with these findings clinical guidelines have been developed to change clinical practice [8,9]. To prevent disability, workers are also advised not to take rest when experiencing back pain, but to stay active [10].

Changes in therapy and general opinion about disability should bring about a change in the incidence of disability. Therefore, changes in management of back pain should be demonstrable in a changing incidence of disability of back pain. Deyo and Cherkin advocate the use of automated databases for this purpose [11,12]. Based on national statistics, there were many reports about an increase of occupational disability as a result of back pain in the seventies and eighties [13-15]. However, it seems that this rise in incidence of occupational disability has stopped somewhere at the end of the eighties. To find time trends in disability as a result of back pain Murphy et al used several databases in the United States and showed that low back pain claim rates decreased by 34% between 1987 and 1995 [16]. From the same databases Hashemi et al found a considerable decrease in disability duration and costs of worker compensation for the period 1988–1996 [17]. The authors link these time trends to interventions directed at the workplace and at the worker with back pain and changes in back pain management. Waddell and Nordlund did an extensive search into figures on occupational disability as a result of back pain to show how social security arrangements could influence neck and back pain in Europe, the USA and Japan [18]. However, they had problems in finding data that could reflect an unbiased estimate of trends of occupational disability as a result of back pain over time. The best they could find was the yearly absolute number of workers on a disability pension as a result of back pain between 1971 and 1997. Since 1993 there appears to be a decrease in this number.

We reported about the incidence of disability as a result of musculoskeletal disorders according to age and gender in the Netherlands in the period 1980–1985 [19]. In comparison with other countries the level of occupational disability has always been high and the criteria for obtaining a benefit rather generous. All employed and self-employed workers are insured against the loss of earning capacity due to impairment resulting from illness. All diseases are accepted as a cause of impairment, whether work-related or not [20]. The increasing incidence of occupational disability in the seventies and eighties is ascribed among others to the broadening of the illness concept. From then on, more subjectively defined complaints and mental disturbances were also accepted as a sufficient cause of impairment leading to entitlement to an occupational disability benefit [21]. Therefore, changes in concepts about occupational disability and management of back pain should result in a concurrent change in occupational disability in the Netherlands. We managed to get data on the incidence of occupational disability in the period 1999–2000 comparable to the data described earlier. However, in the time period between 1985 and 1999 changes occurred in the working population that is insured against occupational disability. More people, especially women, got onto the labor market and became part of the working population that is insured against occupational disability. This change has to be taken into account when data of the two time periods are compared.

Therefore, the objective of our study was to find out if changes occurred in the incidence of occupational disability as a result of back pain in the time period between 1985 and 2000, taking into account changes in the population at risk.

## Methods

In the Netherlands all workers are insured against loss of earnings due to occupational disability. Occupational disability is defined as the loss of earning capacity as a result of the inability to perform work tasks due to disease or infirmity. After 52 weeks of sick leave a worker can claim an occupational disability benefit. About 17% of claims are made by self employed workers or those that don't have a previous working experience. The benefit is only granted after a disability evaluation, which includes a health examination by an insurance physician. The physician makes a diagnosis as a result of this examination or gathers additional information from treating physicians. Therefore, the diagnosis of the disease from which the occupational disability resulted has been backed up by several physicians and can be considered to have at least some kind of validity in contrast to for instance self report.

Data on diagnosis of occupational disability as a result of low back pain according to age and gender were obtained from the bureau of statistics of the Industrial Insurance Administration Office: GAK that was responsible for gathering data on social security for the period 1980–1985. These diagnoses were coded according to the International Classification of Diseases 9th revision [22].

Data for the time period 1999–2000 were obtained from the successor to GAK: LISV, the National Institute of Social Insurance that in this time-period was responsible for gathering these data. Diagnoses were coded according to a classification system that was derived from the International Classification of Diseases 10th revision. Coding in both time frames was done based on medical files at the offices of the social insurance offices by specially trained personal.

To make data comparable we converted all diagnosis codes to the ICD-10 coding system, using an algorithm that ensured comparability of diagnoses (see Additional file 1). The diagnosis back disorders consists of 5 major categories that are all divided into subcategories. For some diagnoses at the third level such as *cervical intervertebral disc diseases *we were not able to disentangle all different sub categories. Therefore, we had to aggregate them at the second level of all *intervertebral disc disorders*. We aggregated diagnostic categories at the lowest level that made comparison possible.

Since people were not subjected to procedures or were required to follow rules of behavior, an approval of the Medical Ethics Committee of the Academic Medical Centre was not required according to the Medical Research Involving Human Subjects Act (WMO).

### Statistical analysis

We calculated average incidence rates per 1000 person years according to age and gender for the specific time periods by dividing the average yearly number of workers that was granted a disability benefit by the average yearly number of insured workers during that period, times 1000. To compare data over the two time periods we calculated incidence-rate ratios (IRR) by dividing the incidence rate of 1999–2000 by the rate of 1980–1985. Therefore, IRRs that are greater than one indicate a rise in occupational disability and IRRs smaller than one a decline. Since occupational disability is much more prevalent in higher age categories, age could easily confound a change in disability between the two time periods. Therefore we standardized the data for age as described by Greenland for person time data. We used the sum of the average populations at risk of the 1985 and 2000 period as the standard. In addition we calculated 95% confidence intervals to take the influence of chance into account [23]. We checked for homogeneity over strata to find out if these adjustments for age were allowed. We compared the time trends in occupational disability due to back disorders to the time trends in occupational disability in general, to be able to account for general time trends, for instance due to changes in economics and legislation. From the IRRs we calculated the percentages of increase or decrease by multiplying the IRR by 100 and then subtracting 100 (Percentage in/decrease = 100%-(IRR*100%).

## Results

There was an increase in the number of workers insured against occupational disability as a result of a large influx into the labor market, especially of women. This resulted also in a shift in the age distribution between the two time periods: fewer workers under 25 years and more workers between 35 and 55 years (Table 1).

In 1999–2000, incidence of occupational disability as a result of all back disorders amounted to 2.02 and 2.14 per 1000 workers per year for men and women respectively (Table 2).

**Figure 1:**
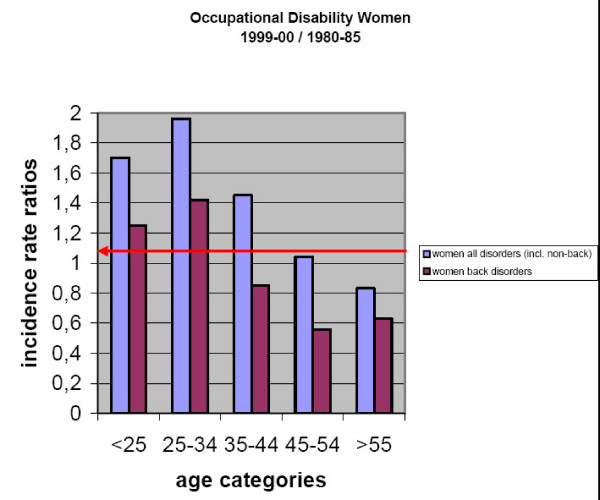
Incidence rate ratios of occupational disability among women due to all back disorders and incidence rate ratios of occupational disability due to any disorder (incl. non-back disorders) for 1999–2000 compared to 1980–1985. Arrow at the rate ratio of 1 indicates no difference between the two time periods.

**Figure 2:**
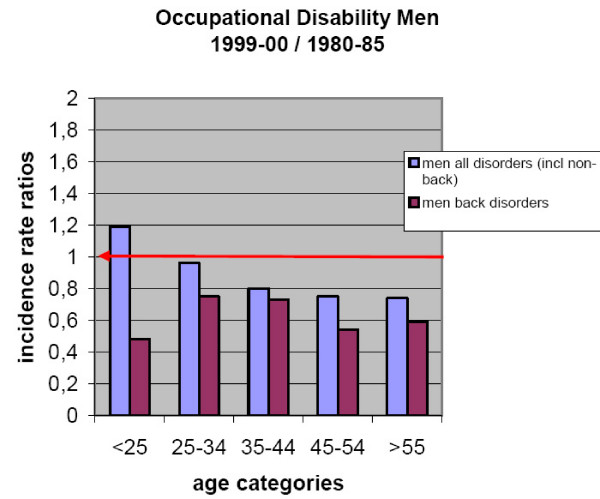
Incidence rate ratios of occupational disability among men due to all back disorders and incidence rate ratios of occupational disability due to any diagnosis (incl. non-back diagnoses) for 1999–2000 compared to 1980–1985. Arrow at the rate ratio of 1 indicates no difference between the two time periods.

In table 3 the incidence rate ratios for the two time periods are given. After adjustment for age differences, it can be deducted from the incidence rate ratios that there was an overall decline in the incidence of occupational disability as a result of back pain of 37% for men and 21% for women. In spite of an increase in several diagnostic categories the overall incidence of back disorders decreased over time. The decrease in occupational disability in general due to any disorder was smaller than that due to the diagnostic category of all back disorders (figure 1 and figure 2).

This decrease was not homogeneous over all age categories. Only for the age strata of the sub categories of *inflammatory back disorders *and *post-laminectomy syndrome *the changes were homogeneous, both in men and women. For women, the changes across strata of the sub-category of *kyphosis *were also homogeneous.

Changes were different between diagnostic categories. For men there was a decrease of 59% for *spondylosis*, 44% for *inflammatory back disorders*, 45% for *intervertebral disc disorders *and 39% for *dorsalgia*. The incidence of other diagnostic categories increased: *other dorsopathies *by 196%, *postlaminectomy syndrome *by 85%. For *kyphosis *there was no significant change.

For women the incidence did not change for *inflammatory back disorders *and *cervicalgia*. The incidence for *spondylosis, intervertebral disc disorders *and *dorsalgia *decreased by 48%, 39% and 21% respectively. The incidence rose for *other dorsopathies *with 136%, for *postlaminectomy syndrome *with 113% and for *kyphosis *with 45%.

Changes varied between age categories. For men the decrease in incidence of all back diagnoses was largest in men under 25 years. Incidence of *cervicalgia *and *other dorsopathies *rose in the younger age categories as opposed to a decrease for older age groups. For women under 35 years the incidence increased for all diagnoses as opposed to a decrease in the older age categories. This was especially the case for *spondylosis*, *dorsalgia *and *cervicalgia*.

## Discussion

We found a decrease in incidence of occupational disability as a result of back disorders between two time periods that were about 17 years apart for men and for older women as expected. Only for women under the age of 35 years the incidence rose. There was a decrease for most diagnostic subcategories especially of anatomical diagnoses like *spondylosis*. The incidence of the category of *other dorsopathies *that mainly consists of *cervicobrachial syndrome *and *post-laminectomy syndrome *increased.

We were able to gather data on occupational disability specific for diagnosis, age and gender that were comparable between two time periods that were sufficiently apart to reflect changes in health care, occupational health, or social security. The assessment procedure of the insurance physician that made the diagnosis was similar between the two time periods. However, the diagnosing physicians of course were not the same. The data reflect large numbers of workers, almost the total labor force of the Netherlands, which increases the reliability of the data. Because we calculated the incidence over periods of time there is less chance that coincidental changes will explain the results. General changes in the management of workers with back pain should be mirrored in these data.

We studied the working population of the Netherlands over an almost twenty year time period. This means that apart from the circumstances in working life and health care also the composition of the cohort changed. Many new workers became part of the working population and many retired. As can be seen from the data, the influx was not equal for gender and age. Nowadays, young people are at school longer and start working life later. Participation of women in the labor market rose with 55%. There is, however, no reason to believe that present workers in the same age category are biologically different from those twenty years ago. Moreover, we think that comparing both cohorts is the best way to study general changes in the incidence of an event. Yet, these changes in the population at risk pose a difficulty in comparing overall incidences. Back pain and especially occupational disability, is strongly related to age. Therefore, changes in age distribution could easily bias differences in incidence. To prevent this bias we adjusted our data to differences in age. However, the changes in incidence of occupational disability were not homogeneous over the age categories, which renders the results of adjustment difficult to interpret. This was especially the case for women, where trends were opposed between older and younger age categories. Overall incidence rate ratios should therefore be interpreted with caution. Inferring a time trend from two points in time leaves the question open as to what happened in between. We did not have data on back pain diagnoses for the time in between. The general trend of occupational disability was an increase in risk during the early eighties and a steady decline thereafter. There is no reason to believe that the time trend for occupational disability as a result of back pain followed a completely different pattern.

In the two time periods different diagnostic classification systems were used, which we had to convert to make them comparable. The current local classification system was entirely based on the ICD-10. Not all diagnostic categories at a three or four digit level were available for the physicians to report a diagnosis. The reporting of diagnosis could therefore be biased towards the main categories. However, we used only main categories for the comparison between the two time periods. Therefore, we think that we succeeded well in converting the diagnostic categories. Moreover the differences between the ICD-10 and ICD-9 are not very big for the chapter on back pain. Even though the ICD-10 seems to be the most comprehensive and widely used coding and classification system used, there is some difficulty in interpreting the codes for the various clinical syndromes. Since diagnostic criteria are lacking we have to make our own inferences about the relation between the codes and the clinical reality.

How can the results be explained? Many things happened in the time period under study. Of course the inferences have to be speculative to a large extent because these are all observational data. However, they can provide interesting clues to the effects of changes in health care. First of all the decrease could be due to a general decrease in occupational disability not specific for back pain. A link has been reported between the rates for occupational disability and unemployment rates, both, in the United States and the Netherlands [21,24]. Apparently, incapacity to work in times of unemployment is more easily attributed to disease then in times of better employment opportunities. In times of a shortage on the labour market it could be that also workers in poorer health get the chance to be employed. Because the economy in most countries in the world has improved substantially in the nineties a decrease in overall occupational disability can be expected. Moreover, politicians have been proposing changes in the social security system constantly in the past twenty years in the Netherlands. Among others the benefit level decreased from mostly 100% to 70% of the latest earned wage in 1987. In general, measures were taken to make it more difficult to obtain a benefit because of occupational disability. This is reflected in an overall general decrease of 18% of occupational disability due to all disorders for men. However, an unexpected increase of 34% for women was found in the time between 1985 and 2000 (Table 3). This higher incidence of disability is explained by the increasing difficulty for women to cope with combined demands from working life and private life, especially in these branches were female workers are predominant such as health care and education [21]. However, the decrease with almost 40% and 20% among men and women respectively, for back pain is much larger. Therefore the change in incidence of disability because of back pain cannot be explained by general trends alone.

It could be that an increase in heavy physical work has led to changed work demands with the result that back pain results more or less easily in occupational disability. The official survey of working conditions does not show such anincrease for the nineties. The percentage of persons that reportheavy physical work is stable at 20% [25].

The trend found could also be due to a change in incidence of back pain. An increase in the 12 month prevalence of back pain has been reported by Palmer et al.[26]. In their study, they did not find an increase in associated disability and therefore they conclude that there was only an increase in reporting of back pain [27]. Other authors report the same incidence of back pain [28,29]. Macfarlane reports a 4% decrease in the 1 month prevalence of back pain during the nineties [30]. Therefore, there is no reason to assume a substantial change in back pain incidence during the nineties.

In the time period under study the management of back pain has changed considerably. The landmark article of the Canadian task force on back pain that was one of the first to advocate a more active approach to prevent occupational disability as a result of back pain was published in 1987 [31]. In 1994 the American Agency on Health Care Policy and Research published a clinical guideline about the management of acute low back pain that followed the same principles [9]. These were followed by similar guidelines in the Netherlands: one for general practitioners in 1996, a general clinical guideline on radiating back pain for all physicians in 1995 and a clinical guideline for occupational physicians in 1999 [32-34]. First, due to these changes, a shift from the specific to the non-specific can be observed. Clinicians do not so much anymore claim to "know" what the cause of the pain is but they acknowledge this non-specific disorder as being uncertain in origin. The shift from anatomical diagnoses to subjective diagnosis is probably the result of this influence. There is evidence that general practitioners follow the guideline on back pain management [35]. However, for some of these developments it is still too early to see a change in outcome. Moreover, it is still unclear to what extent these guidelines have been implemented. This changed view on management of back pain was also successfully introduced to the public in Australia with a change in public opinion as a result [36]. However, even though there are occasional reports in the newspapers there is no organized campaign to change views of the general public in the Netherlands.

Also, in the field of occupational health many things changed. An obligation for all employers to provide occupational health services was introduced in 1996–1998. Especially the obligation to provide rehabilitation by occupational physicians for all workers on sick leave could possibly influence occupational disability. However, it has been shown that early occupational health management was only effective if the guidelines were implemented thoroughly [37,38].

In the literature only few articles report on time trends in occupational disability as a result of back pain [16,17]. Both cover the same short time period of 8 years for which they could demonstrate a decline in the rate of occupational disability as a result of back pain. We covered a larger window of time and could adjust the results for differences in age and gender. We found comparable results for men and older women. Because in previous studies data were not divided according to gender it is unclear if the same trend was present in their material.

It is noteworthy that pain syndromes of the upper extremity such as cervicobrachial disorder and cervicalgia increased in spite of the general trend. These disorders were not covered by practice guidelines in this period. A start has been made with diagnostic guidelines for the work-relatedness of upper extremity disorders [39] and a guideline for the management of return to work for occupational physicians [40].

More research is needed to corroborate these results. Some authors propose to examine trends of processes of care over time or the investigation of duration of functional disability in patients unrelated to work [41]. Experiments with the introduction and better implementation of guidelines could also provide evidence on an effect of improved back pain management on long-term occupational disability. However, none of these studies are easy to perform because data are not available or a long follow-up is needed. Close monitoring of more detailed occupational disability data will also in the future provide leads to the effects of health care on occupational disability.

## Conclusion

From this study, it can be concluded that since 1985 the incidence of occupational disability as a result of back pain decreased more than could be expected from macro-economic developments alone. It is likely that this trend is caused by changes in management of back pain patients. However this decrease could not be demonstrated for younger female workers where incidence rose. Within the categories of back disorders there was a shift from specific to non-specific diagnoses and an increase in post-laminectomy syndrome. These trends deserve further investigation.

## Competing interests

The author(s) declare that they have no competing interests.

## Authors' contributions

IS carried out part of the data processing and drafted the manuscript. JV was responsible for the design of the study, performed the statistical analysis and helped to draft the manuscript. FP gathered the data and helped to draft the manuscript. DK assisted in data analysis and helped to draft the manuscript. All authors read and approved the final manuscript.

## Pre-publication history

The pre-publication history for this paper can be accessed here:



## Figures and Tables

**Table 1:** Average number of insured workers according to age and gender for 1980–1985 and 1999–2000.

Average yearly number of insured workers	< 25 y	25–34 y	35–44 y	44–55 y	>55 y	total
Men 1980–1985	853,000	934,000	762,000	528,000	325,000	3,402,000
Men 1999–2000	624,422	1,027,492	1,001,719	845,005	322,776	3,821,414
						
Women 1980–1985	766.833	465.333	341.000	199.833	88.500	1.861.500
Women 1999–2000	593,456	872,142	739,038	538,698	145,804	2,889,137

**Table 2:** Incidence of occupational disability as a result of back disorders averaged over 1999–2000 per 1000 person-years

***Incidence Men 1000 py 1999–2000***	< 25 y	25–34 y	35–44 y	44–55 y	>55 y	total
*M40–43 Deforming dorsopathies*						
M40–43 Kyphosis and other dp	0.05	0.12	0.17	0.21	0.32	0.16
						
*M45–49 Spondylopathies*						
M45–46 Inflammatory dorsopathies	0.01	0.04	0.04	0.06	0.06	0.04
M47–49 Spondylosis and other sp	0.01	0.05	0.13	0.35	0.98	0.21
						
*M50–54 Other dorsopathies*						
M50–51 Dis of the IVD	0.04	0.44	0.96	1.26	1.47	0.78
M53 Other dorsopathies	0.01	0.08	0.18	0.29	0.63	0.19
M54 Dorsalgia	0.07	0.36	0.68	0.77	0.92	0.54
M54.2 Cervicalgia	0.01	0.03	0.08	0.09	0.12	0.06
M 96.1 Postlaminectomy syndrome	0.01	0.04	0.06	0.07	0.08	0.05
						
Total	0.21	1.15	2.30	3.10	4.57	2.02

***Incidence 1000 py Women 1999 2000***	< 25	25–34	35–44	44–55	>55	total

*M40–43 Deforming dorsopathies*						
M40–43 Kyphosis and other dp	0.07	0.13	0.15	0.18	0.30	0.14
						
*M45–49 Spondylopathies*						
M45–46 Inflammatory dorsopathies	0.01	0.03	0.03	0.04	0.04	0.03
M47–49 Spondylosis and other sp	0.02	0.05	0.11	0.36	0.87	0.16
						
*M50–54 Other dorsopathies*						
M50–51 Disorders of the intervertebral disc	0.09	0.41	0.82	1.27	1.27	0.65
M53 Other dorsopathies	0.03	0.17	0.26	0.47	0.81	0.25
M54 Dorsalgia	0.25	0.79	0.80	1.10	1.30	0.76
M54.2 Cervicalgia	0.03	0.09	0.11	0.16	0.18	0.10
M 96.1 Postlaminectomy syndrome	0.01	0.02	0.05	0.07	0.04	0.04
						
Total	0.52	1.71	2.32	3.66	4.81	2.14

**Table 3:** Incidence rate ratios for occupational disability as a result of back disorders, dividing incidence rates for the period 1999–2000 by those for 1980–1985; adj IRR = for age differences adjusted incidence rate ratio, 95%CI = 95% confidence interval

**Men**	< 25 y	25–34 y	35–44 y	44–55 y	>55 y	tot IRR	adj IRR	95% CI
Categories of disorders								
*M40–43 Deforming dorsopathies*								
M40–43 Kyphosis and other dorsopathies	0.44	0.88	1.22	1.18	1.59	1.10	1.05	0.98–1.11
								
*M45–49 Spondylopathies*								
M45–46 Inflammatory dorsopathies	0.48	0.59	0.47	0.59	0.71	0.62	0.56	0.50–0.62
M47–49 Spondylosis and other spondylopathies	0.61	0.59	0.51	0.35	0.42	0.45	0.41	0.40–0.43
								
*M50–54 Other dorsopathies*								
M50–51 Disorders of the intervertebral disc	0.31	0.61	0.60	0.49	0.53	0.62	0.55	0.53–0.56
M53 Other dorsopathies	6.97	7.78	3.55	1.97	3.30	3.37	2.96	2.71–3.23
M54 Dorsalgia	0.49	0.74	0.74	0.52	0.51	0.69	0.61	0.59–0.63
M54.2 Cervicalgia	2.46	1.81	1.28	0.44	0.29	0.67	0.60	0.55–0.66
M 96.1 Postlaminectomy syndrome	2.05	2.02	1.73	1.74	2.32	2.12	1.85	1.62–2.14
								
*All back disorders*	0.48	0.75	0.73	0.54	0.59	0.70	0.63	0.62–0.64
*All disorders (incl. non-back disorders)*	1.19	0.96	0.80	0.75	0.74	0.88	0.82	0.81–0.82

**Women**	< 25 y	25–34 y	35–44 y	44–55 y	>55 y	tot IRR	adj IRR	95% CI

Categories of disorders								
*M40–43 Deforming dorsopathies*								
M40–43 Kyphosis and other deformities	1.03	1.61	1.73	1.40	1.54	1.59	1.45	1.32–1.60
								
*M45–49 Spondylopathies*								
M45–46 Inflammatory dorsopathies	1.15	1.06	0.78	1.07	1.09	1.20	0.99	0.80–1.21
M47–49 Spondylosis and other spondyopathies	1.39	1.41	0.69	0.45	0.42	0.70	0.52	0.48–0.56
								
*M50–54 Other dorsopathies*								
M50–51 Disorders of the intervertebral disc	0.85	0.91	0.62	0.51	0.49	0.83	0.61	0.58–0.63
M53 Other dorsopathies	3.68	4.49	2.17	1.76	2.59	3.23	2.36	2.12–2.69
M54 Dorsalgia	1.30	1.47	0.88	0.44	0.63	1.02	0.79	0.76–0.82
M54.2 Cervicalgia	4.63	2.76	1.12	0.53	0.46	1.28	0.95	0.84–1.06
M 96.1 Postlaminectomy syndrome	1.82	2.52	1.89	2.39	1.82	2.81	2.13	1.64–2.77
								
*All back disorders*	1.25	1.42	0.85	0.56	0.63	1.05	0.79	0.78–0.81
*All disorders (incl. non-back disorders)*	1.70	1.96	1.45	1.04	0.83	1.63	1.34	1.33–1.35
